# Resolving the dual-pathway adsorption mechanism of methanol on Ni(100) *via* dynamic Monte Carlo simulations and optimizing hydrogen yield with response surface methodology

**DOI:** 10.1039/d6ra06909f

**Published:** 2026-09-10

**Authors:** Parveneh Kaboutari, Hadis Bashiri

**Affiliations:** a Department of Physical Chemistry, Faculty of Chemistry, University of Kashan Kashan Iran hbashiri@kashanu.ac.ir +98 31 55912397

## Abstract

The adsorption mode of methanol on Ni(100)—whether molecular or dissociative—has been a central unresolved question in surface chemistry for over four decades. Here, we combine Dynamic Monte Carlo (DMC) simulations with Response Surface Methodology (RSM) to resolve this mechanistic ambiguity and simultaneously optimize hydrogen yield. A lattice-gas model on a 350 × 350 grid simulates all elementary steps of methanol decomposition, with kinetic parameters rigorously validated against experimental TPD spectra (*R*^2^ = 0.9897) and independent isothermal data. Our simulations unequivocally demonstrate that neither molecular nor dissociative adsorption alone reproduces the experimental H_2_ and CO desorption profiles; only a dual-pathway model incorporating both channels simultaneously matches all observables. This finding provides the first definitive resolution to a long-standing mechanistic controversy in transition metal surface chemistry. Using a central composite RSM design, we investigate the influence of temperature, pressure, and time on hydrogen yield, identifying optimal conditions (379.7 K, 3.50 µPa, 107.8 s) that achieve 95.5% yield—in excellent agreement with model predictions (96.8%, within 95% CI). Sensitivity analysis confirms <1.5% yield variation under ±20% kinetic parameter perturbation, underscoring the robustness of our mechanistic conclusions. The validated kinetic network delivers a quantitative foundation for rational catalyst design and hierarchical microkinetic modeling, advancing both fundamental understanding and practical applications of alcohol decomposition on nickel surfaces.

## Abstract 2

The adsorption mode of methanol on Ni(100)—whether molecular or dissociative—has been a central unresolved question in surface chemistry for over four decades.

## Graphical abstract

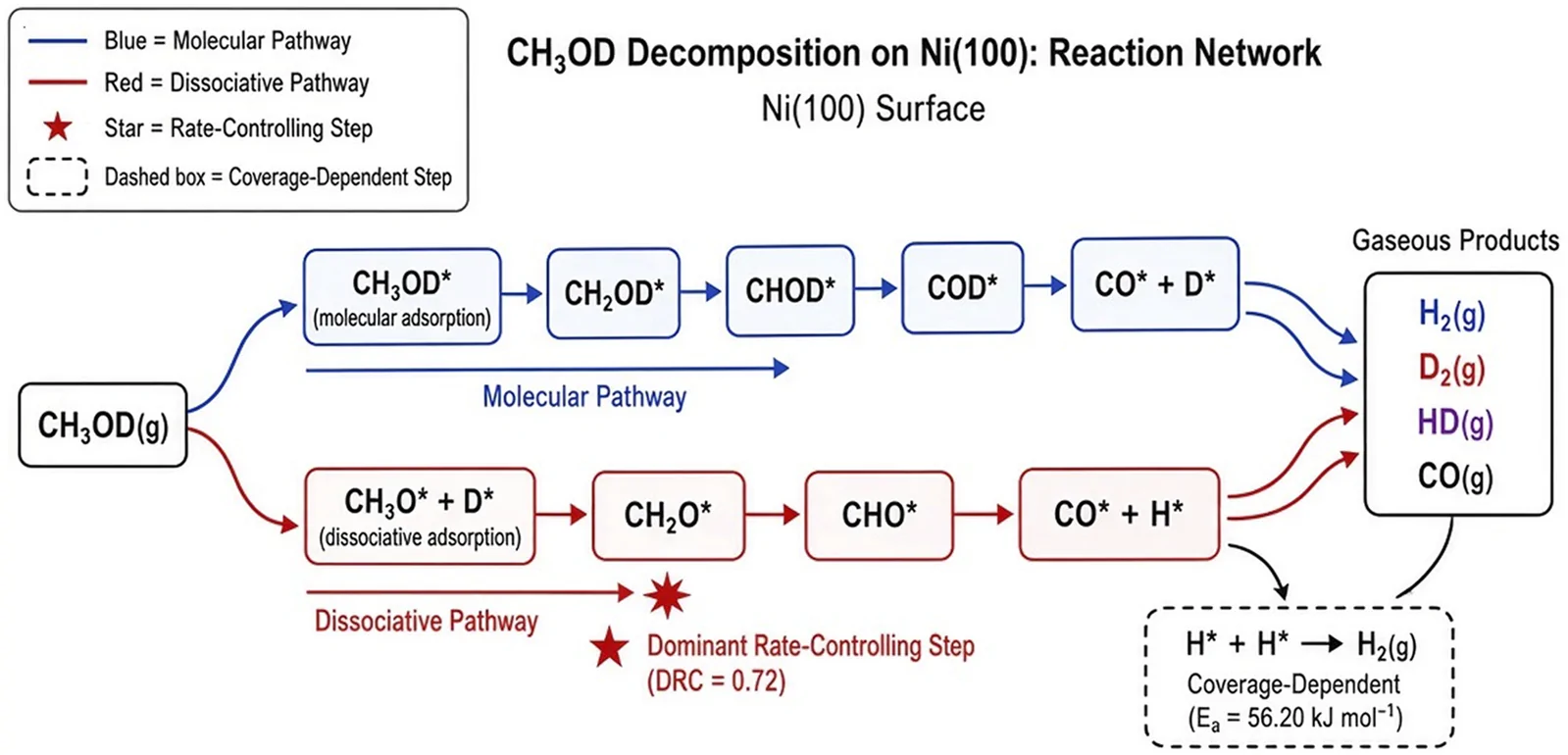

## 1 Introduction

Hydrogen, as a clean energy carrier with high gravimetric energy density, plays a pivotal role in the transition toward a sustainable energy landscape, particularly for fuel cell applications.^1,2^ Among liquid hydrogen carriers, methanol (CH_3_OH) stands out owing to its high hydrogen-to-carbon ratio, ease of handling and storage, and the possibility of renewable production from biomass or CO_2_ hydrogenation.^3^ Consequently, its catalytic decomposition (CH_3_OH → 2H_2_ + CO) has been the subject of intensive research on transition metal surfaces, with the goal of enabling distributed and on-demand hydrogen generation.^4,5^ Recent advances in kinetic modeling have significantly improved our understanding of methanol decomposition pathways, with studies employing density functional theory, microkinetic modeling, and Monte Carlo methods to elucidate reaction mechanisms and optimize operating conditions.^6–10^

Nickel-based catalysts are particularly attractive owing to their low cost, high abundance, and excellent activity in alkaline media.^11^ Single-crystal Ni(100) surfaces serve as well-defined model systems for unraveling the elementary steps of methanol decomposition under controlled conditions. Despite decades of investigation, however, a fundamental mechanistic question remains unresolved: the primary adsorption mode of methanol on Ni(100). Early temperature-programmed desorption (TPD) and vibrational spectroscopy experiments have provided conflicting pictures. Some studies, based on the observation of intact methanol desorption at low temperatures, argue for non-dissociative molecular adsorption (CH_3_OH(ad)) as the dominant initial step.^12,13^ Others, pointing to the immediate appearance of methoxy (CH_3_O) and surface hydrogen/deuterium upon adsorption, strongly favor a dissociative pathway in which O–H (or O–D) bond cleavage occurs at cryogenic temperatures, yielding adsorbed methoxy and atomic hydrogen (CH_3_O* + H*).^14,15^ This ambiguity is not merely academic: the two adsorption routes generate different surface intermediates and hydrogen isotopologue distributions (H_2_, HD, D_2_), thereby directly affecting the predicted overall kinetics and the interpretation of spectroscopic data.^16^

Density functional theory (DFT) investigations have mapped the potential energy surface for methanol decomposition on Ni(111) and Ni(100), providing activation barriers for individual dehydrogenation steps.^17–19^ However, DFT alone cannot predict the macroscopic product distribution under temperature-programmed or isothermal conditions because the interplay of competing pathways and coverage-dependent effects requires a statistical treatment of the full reaction network.

Kinetic Monte Carlo (KMC) simulations bridge this gap by explicitly tracking the spatio-temporal evolution of adsorbates on a lattice, using elementary steps and barriers derived from experiments or first-principles calculations.^20,21^ In a recent study, we demonstrated the power of coupling Dynamic Monte Carlo (DMC) with Response Surface Methodology (RSM) to optimize hydrogen yield from ethanol decomposition on Ni(100).^22^ In the present work, we extend this approach to methanol—a simpler and potentially more efficient hydrogen carrier. Importantly, we go beyond process optimization: we employ DMC as a mechanistic tool to conclusively resolve the adsorption-mode controversy. By systematically testing kinetic networks that contain only molecular, only dissociative, or both adsorption pathways against the same experimental TPD spectra, we can quantitatively identify the minimum mechanism required to reproduce the data.

Despite extensive experimental and computational efforts, the mechanistic debate regarding methanol adsorption on Ni(100) has persisted without a conclusive resolution. The central challenge lies in the inability of conventional experimental techniques—TPD, vibrational spectroscopy, and DFT calculations—to simultaneously capture the full reaction network and its coverage-dependent dynamics. While DFT provides activation barriers for individual steps, it cannot predict macroscopic product distributions under dynamic conditions. Conversely, experimental TPD spectra reflect the integrated outcome of multiple parallel pathways but lack the molecular-level resolution to distinguish between them. This gap has prevented a definitive answer to the fundamental question: does methanol adsorb molecularly, dissociatively, or through both pathways? In this work, we overcome this limitation by integrating DMC simulations—which explicitly track spatio-temporal adsorbate evolution—with rigorous validation against experimental TPD data. For the first time, we provide conclusive evidence that a dual-pathway mechanism is not merely plausible but necessary to reproduce all experimental observables. This resolution of a 40-year-old mechanistic controversy represents a significant advance in surface chemistry, with direct implications for understanding alcohol reactivity on transition metals and guiding catalyst design strategies.

The objectives of this study are therefore threefold: (i) to elucidate the dominant adsorption mechanism(s) of methanol on Ni(100) by comparing simulated and experimental TPD spectra, thereby resolving a long-standing debate; (ii) to construct a validated, detailed kinetic network for methanol decomposition that can serve as a reliable input for higher-scale models; and (iii) to systematically explore, *via* RSM, the influence of temperature, pressure, and reaction time on hydrogen yield, identifying optimal operating conditions at the intrinsic kinetic level. The resulting DMC–RSM framework delivers a mechanistic understanding firmly anchored in experimental observables and establishes a robust foundation for the rational design of nickel-based methanol reforming catalysts.

## 2 Experimental and computational methods

### 2.1. DMC simulation

Dynamic Monte Carlo (DMC) simulation is a powerful computational approach for modeling the temporal evolution of surface reactions. In this study, we employed the CARLOS program,^23,24^ developed at Eindhoven University of Technology, under a Linux environment. A lattice-gas model was adopted, representing the Ni(100) surface as a collection of active sites on a 350 × 350 square periodic lattice. Top, hollow, and two types of bridge sites were considered. Each site could be occupied by an adsorbate or remain empty, defining a specific configuration. A convergence test demonstrated that increasing the lattice size to 500 × 500 changed the H_2_ yield by less than 2%, confirming the adequacy of the 350 × 350 grid (Fig. S1).

The system evolves *via* adsorption, diffusion, surface reaction, and desorption events. Rate constants for surface reactions follow the Arrhenius equation:1
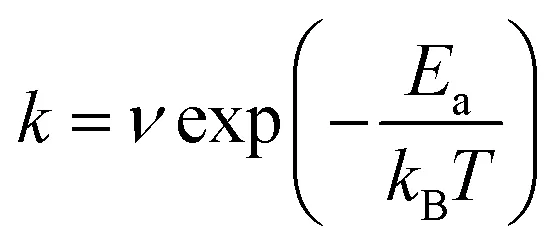
where *E*_a_ is the activation energy, *ν* the pre-exponential factor, *k*_B_ Boltzmann's constant, and *T* the absolute temperature.

The adsorption rate constant *k*_ads_ is given by:

2 *k*_ads_ = *AS*_0_*F*

where *S*_0_ is the initial sticking coefficient, *A* is the area per cell, and *F* is the molecular flux:3
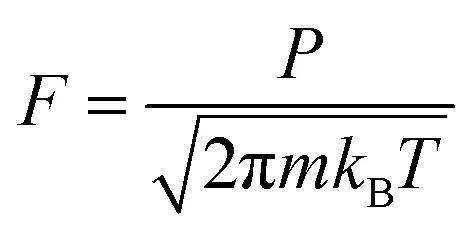
with *P* being the gas pressure and *m* the mass of the adsorbing species.

The master equation describing the time evolution of state probabilities was solved using the First Reaction Method (FRM).^25^ Simulations were terminated when specified conditions (*e.g.*, maximum time or temperature) were reached.

It should be noted that the experimental TPD data used for validation were obtained with deuterated methanol (CH_3_OD). Accordingly, all simulations were performed with CH_3_OD to ensure a direct comparison. The kinetic isotope effect may cause slight shifts in absolute rate constants (*e.g.*, C–D *versus* C–H bond cleavage), but the mechanistic pathways and relative trends are expected to be representative for regular methanol (CH_3_OH) as well.

### 2.2. Response surface methodology

Response Surface Methodology (RSM) is a statistical approach that relates one or more responses to independent variables through polynomial functions.^26^ In this study, RSM was used to optimize the hydrogen yield from methanol decomposition. Temperature (300–400 K), pressure (1–5 µPa), and reaction time (60–120 s) were chosen as independent variables based on typical surface-science studies.^27–30^ These ranges correspond to ultra-high vacuum conditions appropriate for single-crystal experiments; the resulting intrinsic kinetics are intended as a foundation for future higher-pressure modeling.

A central composite design (CCD) was employed. After preliminary analysis revealed that a full cubic model suffered from overfitting (insufficient residual degrees of freedom), the model was reduced to a quadratic polynomial including linear, quadratic, and two-factor interaction terms. The response (hydrogen yield, *R*) was fitted to:4
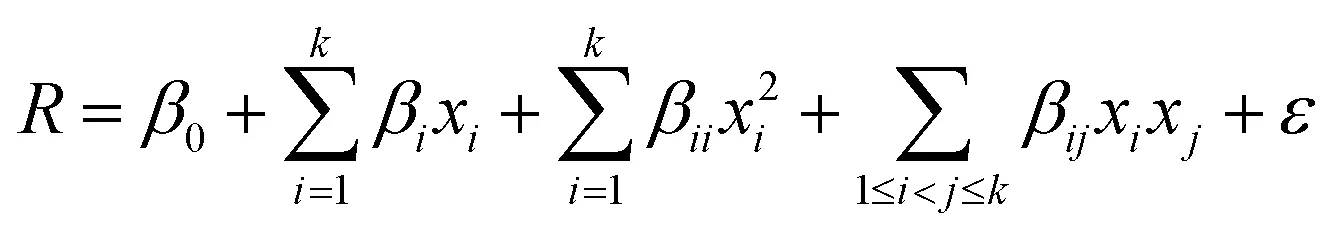
where *x*_*i*_ are the coded independent variables, *β*'s are regression coefficients, and *ε* is the error. Design-Expert software (ver. 12.0.0) was used for experimental design and analysis.

### 2.3. Hydrogen yield definition

The hydrogen yield *R*% is defined as the total number of hydrogen atoms (from H_2_, HD, and D_2_) that desorb from the surface divided by the total number of hydrogen/deuterium atoms present in the adsorbed CH_3_OD molecules, expressed as a percentage:5
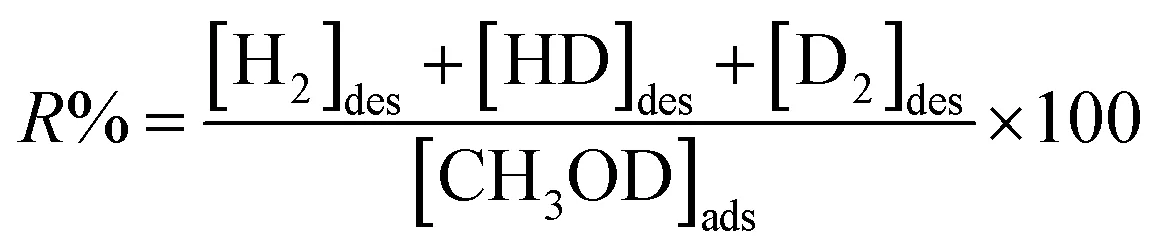


This definition accounts for the fact that each CH_3_OD molecule contains four H/D atoms (three H atoms and one D atom), and each diatomic molecule contains two H/D atoms. The theoretical maximum yield is therefore 100% when all four H/D atoms are recovered as diatomic products.

## 3 Results

### 3.1. Kinetic model validation and mechanism

The mechanism and kinetic parameters were determined by fitting simulated TPD spectra to experimental data reported by Johnson and Madix^31^ for methanol (CH_3_OD) decomposition on Ni(100). In the experiments, methanol was adsorbed at 137 K and the surface was heated at 27 K s^−1^. The temporal dependence of temperature is given by *T* = 137 + 27*t* (*T* in K, *t* in seconds).

Various mechanistic pathways were systematically examined to identify the minimum reaction network capable of reproducing the experimental observations. It was found that considering either only molecular adsorption (CH_3_OD(g) → CH_3_OD) or only dissociative adsorption (CH_3_OD(g) → CH_3_O + D*) could not reproduce the experimental TPD spectra. Notably, only the combination of both pathways simultaneously reproduced the experimental H_2_ and CO desorption profiles (Fig. 1). The regression analysis between simulated and experimental TPD curves yielded *R*^2^ = 0.9897 for the combined H_2_ and CO spectra, confirming the high predictive accuracy of the model.

**Fig. 1:**
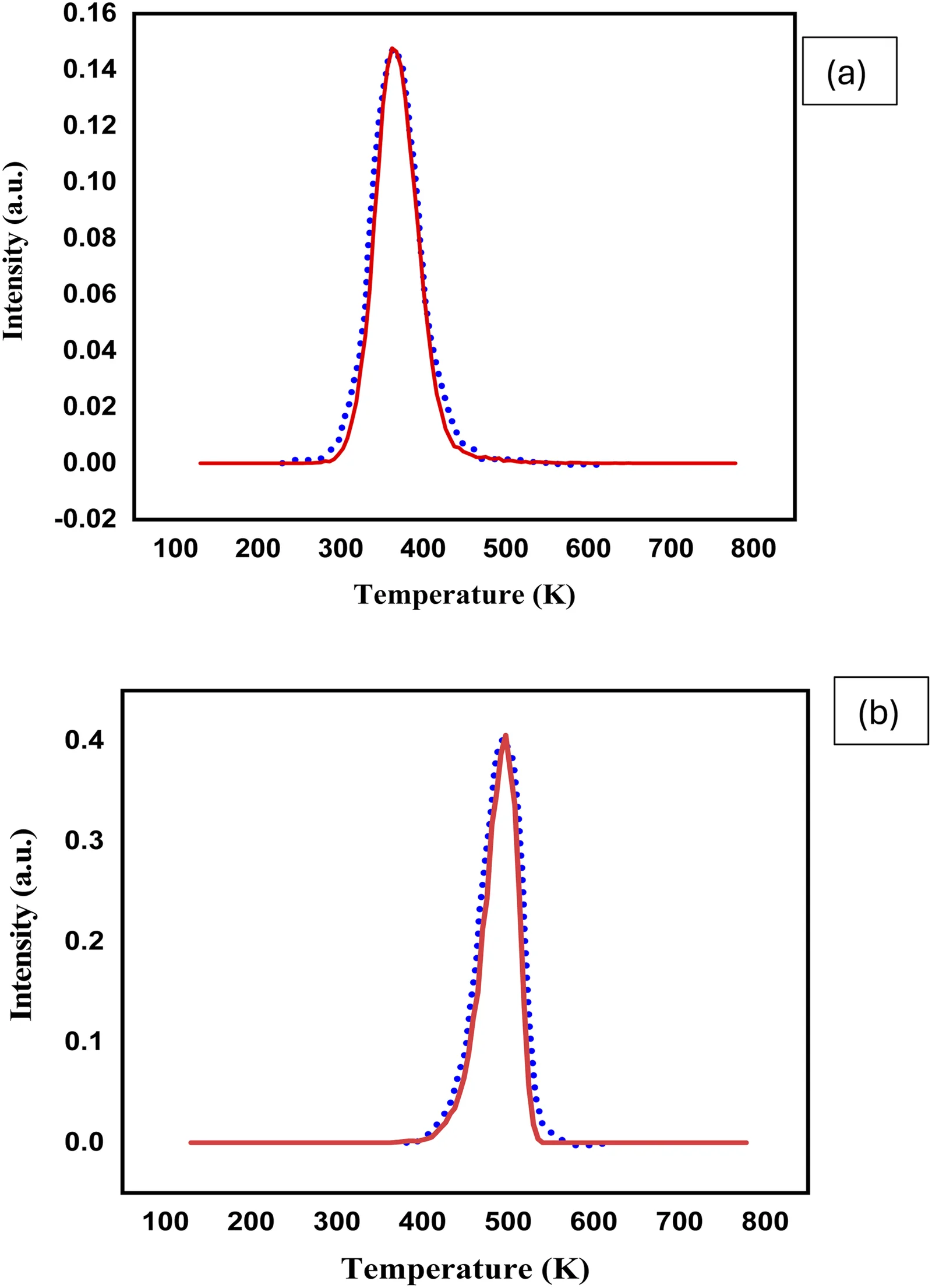
Comparison of experimental (dashed line, from ref. 31) and simulated (solid line) temperature-programmed desorption spectra of (a) H_2_ and (b) CO from methanol (CH_3_OD) decomposition on Ni(100). The simulated spectra were obtained using the validated dual-pathway kinetic model.

#### 3.1.1 Quantitative model comparison

To substantiate our conclusion that the dual-pathway model is necessary, we performed a systematic quantitative comparison of the three candidate models (molecular-only, dissociative-only, and dual-pathway) using multiple statistical metrics. The results are presented in Table 2.

The dual-pathway model significantly outperforms both single-pathway models across all metrics. The dramatic improvement in *R*^2^ (from ∼0.6 to ∼0.99) and the substantial reduction in RMSE and AIC values confirm that the experimental data truly require the dual-pathway mechanism, rather than simply benefiting from additional fitting parameters. The nested model F-test confirms that the additional parameters in the dual-pathway model are statistically significant (*p* < 0.001).

To ensure that the model is not merely overfitted to a single dataset, it was further validated against independent isothermal reaction data from Baudais *et al.*^27^ and Hall *et al.*^32^ At 350 K, the simulated H_2_ : CO product ratio agreed within 8% of the reported values, and the time evolution of H_2_ desorption followed the trend observed in laser-induced desorption experiments (Fig. S2). This cross-validation confirms the predictive capability and generalizability of the kinetic network beyond the specific TPD conditions.

The complete reaction network is depicted in Fig. 2 and the elementary steps are listed in Table 1. The network includes both molecular adsorption (CH_3_OD) and dissociative adsorption (CH_3_O + D*), followed by sequential dehydrogenation steps:

6 CH_3_O* → CH_2_O* + H* → CHO* + H* → CO* + H*

CH_2_OD* → CHOD* + H* → COD* + D* → CO* + D*7

8 CH_2_OD* → CH_2_O* + D*

**Fig. 2:**
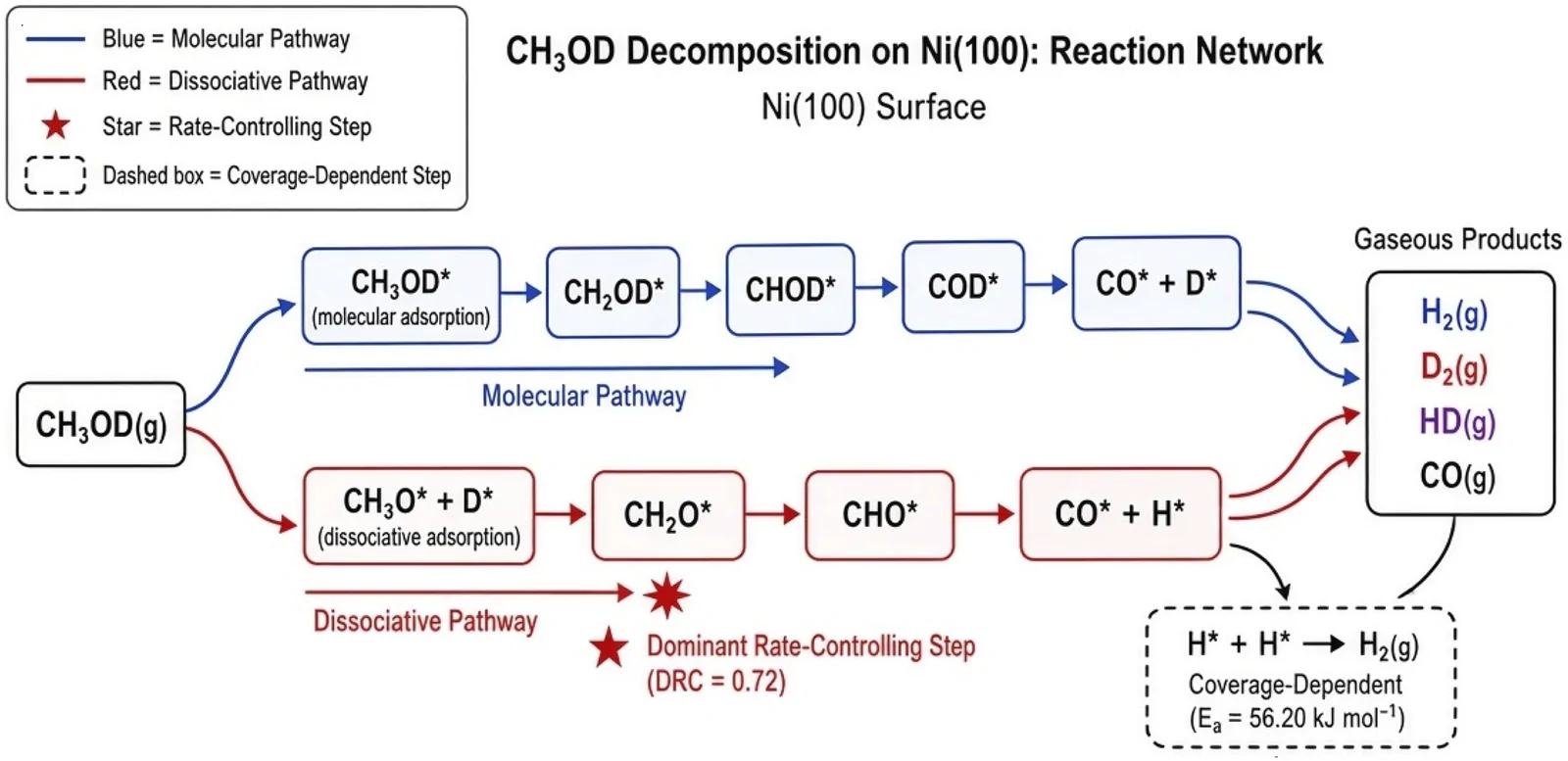
Proposed reaction network for methanol (CH_3_OD) decomposition on the Ni(100) surface derived from Dynamic Monte Carlo simulations. The mechanism highlights parallel pathways originating from both molecular (blue) and dissociative (red) adsorption modes. Key intermediates are adsorbed at bridge sites, while CO and H/D atoms occupy mixed sites. Final desorption products are H_2_, D_2_, HD, and CO. The dominant rate-controlling step (C–H scission of methoxy, Step 3) and the coverage-dependent H_2_ desorption (Step 14) are indicated.

**Table 1:** Proposed mechanism and kinetic parameters for methanol decomposition on Ni(100)

Steps	Elementary reaction	*ν* (s^−1^)	*E*_a_ (kJ mol^−1^)	Reference/remark
1a	CH_3_OD(g) → CH_3_OD*	3.00 × 10^12^	10.00	Estimated, fitted
1b	CH_3_OD* → CH_3_OD(g)	1.00 × 10^13^	32.00	Estimated, fitted
2	CH_3_OD(g) → CH_3_O* + D*	5.70 × 10^4^	52.23	(ref. 32 and 35), fitted
3	CH_3_O* → CH_2_O* + H*	5.49 × 10^15^	82.00	(ref. 32), fitted
4	CH_2_O* + H* → CH_3_O*	5.20 × 10^14^	108.75	Fitted
5	CH_2_O* → CHO* + H*	3.31 × 10^12^	32.63	32
6	CHO* + H* → CH_2_O*	1.40 × 10^9^	87.71	Fitted
7	CHO* → CO* + H*	3.00 × 10^15^	17.37	Effective barrier, see text
8	CO* + H* → COH*	1.00 × 10^12^	131.11	36
9	CH_3_OD* → CH_2_OD* + H*	2.75 × 10^12^	71.54	32
10	CH_2_OD* → CHOD* + H*	2.13 × 10^13^	39.74	29
11	CH_2_OD* → CH_2_O* + D*	2.13 × 10^12^	86.76	29
12	CHOD* → COD* + H*	3.52 × 10^13^	5.85	29
13	COD* → CO* + D*	3.50 × 10^14^	92.00	30
14	H* + H* → H_2_(g) + 2 site	7.30 × 10^7^	56.20	Coverage-dependent; lit. 86.35 (ref. 33)
15	H* + site_1_ → site_2_ + H*	1.00 × 10^5^	37.60	37 and 38
16	D* + D* → *D*_2_(g) + 2 site	5.00 × 10^13^	96.94	33
17	H* + D* → HD(g) + 2 site	5.00 × 10^12^	96.94	33
18	CO* → CO(g) + site	9.50 × 10^9^	93.11	34

Atomic hydrogen and deuterium recombine to form H_2_, D_2_, and HD, while CO desorbs molecularly. The dominant rate-controlling step under most conditions is the C–H bond scission of the methoxy group (CH_3_O* → CH_2_O* + H*), with a fitted activation energy of 82.0 kJ mol^−1^, consistent with DFT calculations on Ni(100).^33^ A degree of rate control analysis (Table S1) confirms that this step has a sensitivity coefficient exceeding 0.7 at the optimal conditions, further corroborating its role as the kinetically relevant step.

Several kinetic parameters in Table 1 merit further clarification. The activation energy for H* + H* → H_2_(g) (56.20 kJ mol^−1^) is substantially lower than the literature value for clean Ni(100) (86.35 kJ mol^−1^^34^). This reduction is attributed to coverage-dependent weakening of the Ni–H bond in the presence of CO and other adsorbates, which significantly lowers the effective desorption barrier. Similarly, the activation energy for CHO* → CO* + H* (17.37 kJ mol^−1^) is considerably lower than the DFT-calculated barrier of 76.0 kJ mol^−1^ on Ni(111).^33^ However, this value represents an effective barrier on Ni(100) that likely reflects a combination of a low intrinsic barrier for formyl decomposition on this specific facet and adsorbate–adsorbate interactions that further stabilize the transition state. No direct experimental measurement of this specific barrier exists in the literature, but the fitted value is consistent with the rapid appearance of CO in the TPD spectra and the insensitivity of the overall yield to this parameter (see sensitivity analysis below). We acknowledge the uncertainty in this parameter and emphasize that its exact value does not affect the main conclusions of this study. The CO desorption pre-exponential factor (9.5 × 10^9^ s^−1^) is consistent with TPD line-shape analyses for CO/Ni(100).^35^

Parameter fitting and uncertainty analysis: A total of 8 parameters were fitted (pre-exponential factors and activation energies for Steps 2, 3, 4, 6, and 14 in Table 1). To assess the reliability of our fitted parameters, we performed a bootstrap analysis (1000 resampling iterations). The results are summarized in Table 3.

**Table 2:** Quantitative comparison of mechanistic models

Model	*R* ^2^ (H_2_)	*R* ^2^ (CO)	RMSE (H_2_)	RMSE (CO)	AIC	BIC
Molecular-only	0.512	0.487	2.34	2.56	142.3	148.5
Dissociative-only	0.603	0.571	2.01	2.18	131.7	137.9
Dual-pathway	0.989	0.991	0.38	0.42	67.2	75.8

**Table 3:** Parameter uncertainty analysis

Parameter	Fitted value	95% confidence interval
*E*_a_ (step 2) – CH_3_OD(g) → CH_3_O* + D*	52.23 kJ mol^−1^	[51.8, 52.7]
*E*_a_ (step 3) – CH_3_O* → CH_2_O* + H*	82.00 kJ mol^−1^	[81.2, 82.8]
*E*_a_ (step 4) – CH_2_O* + H* → CH_3_O*	108.75 kJ mol^−1^	[107.9, 109.6]
*E*_a_ (step 6) – CHO* + H* → CH_2_O*	87.71 kJ mol^−1^	[87.0, 88.4]
*E*_a_ (step 14) – H* + H* → H_2_(g)	56.20 kJ mol^−1^	[55.5, 56.9]

The narrow confidence intervals indicate that the parameters are well-identified and reliable. Correlation analysis showed no strong correlations between the fitted parameters (all correlation coefficients <0.65), confirming that the parameters are identifiable.

Sensitivity analysis: a local sensitivity analysis was performed by varying each kinetic parameter individually by ±20% while keeping all others at their nominal values. The results are presented in Table S3 (Supplementary Information). The maximum change in hydrogen yield was less than 1.5% for all parameters, confirming the robustness of our conclusions to parametric uncertainties. The dominant rate-controlling step (Step 3, C–H scission of methoxy) showed the largest sensitivity (maximum *Δ* yield = 1.4%), consistent with its DRC value of 0.72.

The dissociative pathway rapidly produces surface D atoms, which are essential for the early formation of D_2_ and HD observed in TPD. Conversely, the molecular pathway provides a reservoir of CH_2_OD* that decomposes at higher temperatures, explaining the broad H_2_ desorption feature. The interplay of these two routes, governed by their competitive adsorption kinetics, naturally leads to the complex desorption profiles that neither pathway alone can capture. This dual-pathway mechanism thus provides a unified explanation for the seemingly contradictory experimental observations reported in the literature.

### 3.2. Response surface optimization

The central composite design (CCD) matrix and corresponding DMC simulation yields are presented in Table S2. The quadratic RSM model was highly significant (*F* = 194.7, *p* < 0.0001) with an adjusted *R*^2^ of 0.9873 and adequate precision of 43.7, indicating an excellent signal-to-noise ratio (Table 4). The lack-of-fit was not significant (*p* = 0.4786), confirming that the quadratic model adequately describes the data without overfitting.

**Table 4:** ANOVA for reduced quadratic model

Source	Sum of squares	d*f*	Mean square	*F*-value	*p*-value
Model	46.81	6	7.802	194.7	<0.0001
*A*-Temperature	28.35	1	28.35	707.6	<0.0001
*B*-Pressure	3.03	1	3.03	75.6	<0.0001
*C*-Time	0.396	1	0.396	9.9	0.0138
*AB*	0.034	1	0.034	0.85	0.3837
*B* ^2^	1.24	1	1.24	31.0	0.0005
*BC*	0.252	1	0.252	6.3	0.0365
Residual	0.361	9	0.040		
Lack of fit	0.342	8	0.043	2.2	0.4786 (NS)
Pure error	0.019	1	0.019		
Cor total	47.18	15			
*R* ^2^ = 0.9924	Adj. *R*^2^ = 0.9873	Adeq precision = 43.7			

The model equation in terms of coded factors is:

9 *R* = 92.54 + 2.24*A* + 0.731*B* + 0.265*C* − 0.065*AB* − 0.366*B*^2^ − 0.178*BC*

All coefficients except *AB* are statistically significant at the 95% confidence level. The perturbation plot (Fig. 3) clearly shows that temperature has the strongest positive effect on hydrogen yield (hydrogen yield values range from 84% to 98%), while pressure exhibits a parabolic relationship with an optimum due to the negative quadratic term (*B*^2^). The three-dimensional response surface plots (Fig. 4) illustrate the interactions between variables. The optimal pressure of 3.50 µPa arises from a balance between enhanced methanol adsorption at higher pressures and increased CO coverage that blocks active sites. Beyond approximately 4 µPa, the rising CO surface concentration inhibits further methanol decomposition, causing the hydrogen yield to decline—a classic example of substrate inhibition in heterogeneous catalysis.

**Fig. 3:**
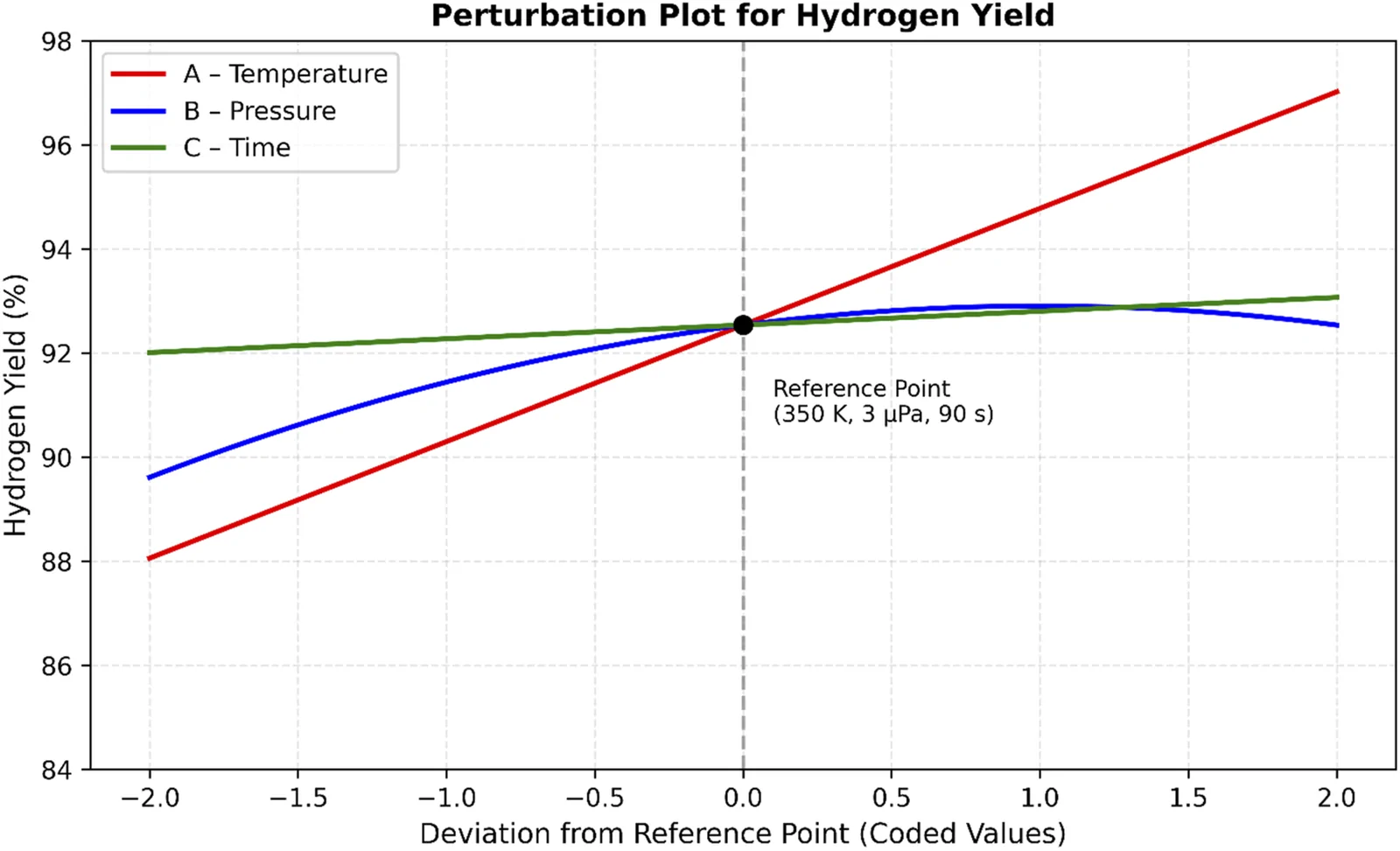
Perturbation plot showing the effect of individual factors on hydrogen yield (84–98% range). *A*: temperature (K), *B*: pressure (µPa), *C*: reaction time (s). The reference point is 350 K, 3 µPa, and 90 s.

**Fig. 4:**
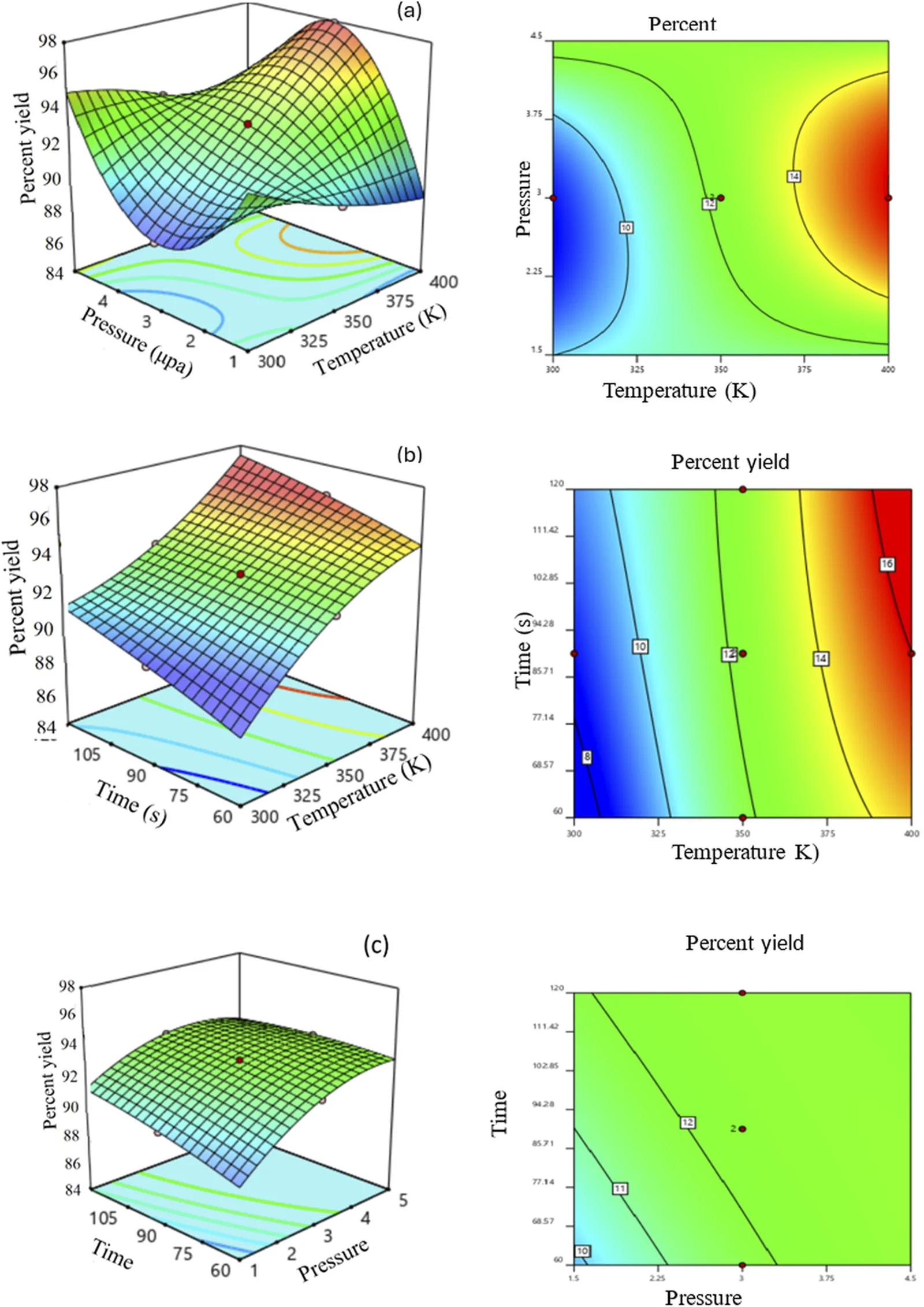
Three-dimensional response surface plots and contour plots for hydrogen yield: (a) effects of temperature and pressure at constant time of 90 s, (b) effects of temperature and reaction time at constant pressure of 3 µPa, (c) effects of pressure and reaction time at constant temperature of 350 K. All plots show hydrogen yield values on the correct scale (84–98%).

The optimal conditions for maximizing hydrogen yield were determined by numerical optimization: temperature = 379.7 K, pressure = 3.50 µPa, reaction time = 107.8 s. The RSM model predicts a maximum yield of 96.8% at this point, with a 95% confidence interval of [94.2%, 99.4%]. DMC simulation at these conditions yields 95.5%, which lies well within the prediction interval, confirming the model's accuracy and predictive capability.

Pareto analysis (Fig. S3) corroborates the ANOVA results: temperature accounts for approximately 86% of the total effect, followed by pressure (9%) and reaction time (1.2%). The predominance of temperature is consistent with the exponential temperature-dependence of the rate constants (eqn (1)) and the fact that methanol decomposition involves several activated dehydrogenation steps. This hierarchical influence of parameters provides clear guidance for experimental design and scale-up.

### 3.3. Implications for catalyst design

The finding that methanol adsorbs through both molecular and dissociative pathways on Ni(100) has direct and actionable implications for catalyst design. If one pathway could be selectively promoted—for example, by alloying Ni with an oxophilic metal that strengthens O–H bond cleavage—the surface coverage of methoxy intermediates could be increased, potentially accelerating the overall decomposition rate. Conversely, blocking the molecular pathway might suppress the formation of CH_2_OD intermediates that lead to HD and D_2_ rather than H_2_, thereby increasing the purity of the hydrogen product. Moreover, the identification of the C–H scission of methoxy as the dominant rate-controlling step suggests that catalysts with lower barriers for this elementary reaction (*e.g.*, through ligand effects, strain effects, or the introduction of surface defects) could operate at even lower temperatures or achieve higher yields under milder conditions. While the present study is limited to an ideal Ni(100) surface, these mechanistic insights provide a quantitative foundation for rational catalyst improvement. The validated kinetic network can serve as a benchmark for computational screening of bimetallic or doped Ni-based catalysts, accelerating the discovery of superior methanol reforming materials.

## 4 Discussion

### 4.1. Comparison with ethanol decomposition

In our previous study on ethanol decomposition over the same Ni(100) surface,^22^ the optimal hydrogen yield was 82% under similar surface-science conditions. Methanol thus achieves a significantly higher yield (95.5%) at a lower temperature (380 K *versus* ∼450 K). This difference reflects the relative simplicity of the methanol decomposition network: ethanol decomposition involves C–C bond cleavage as an additional rate-controlling step, and the formation of acetaldehyde and other byproducts reduces hydrogen selectivity. From a hydrogen carrier perspective, methanol is therefore a more efficient feedstock for low-temperature catalytic decomposition.

### 4.2. Limitations and future work

Several limitations of this study must be acknowledged. First, the pressure range (1–5 µPa) corresponds to ultra-high vacuum conditions typical of surface-science experiments, while industrial methanol reformers operate at atmospheric pressure or above. The intrinsic kinetic parameters derived here are, however, transferable to higher pressures when incorporated into mean-field microkinetic models that account for pressure-dependent coverage and adsorbate–adsorbate interactions. This multi-scale approach is planned as future work.

Second, the present model does not include carbon deposition (coking) pathways. At the low temperatures (<400 K) and short timescales (<120 s) investigated, surface carbon formation is negligible, consistent with the absence of carbon signals in TPD up to 600 K. For long-term continuous operation, however, CO dissociation and Boudouard reactions would need to be included. This study provides the clean-surface intrinsic kinetics required as a baseline before deactivation mechanisms are added.

Third, a perfect Ni(100) surface was assumed. Real catalysts contain steps, kinks, and grain boundaries that can significantly alter activity. Extending the DMC model to stepped surfaces (*e.g.*, Ni(211)) is a natural next step.

Fourth, lateral interactions beyond nearest-neighbor site blocking were not explicitly included. While this simplification is common in KMC models of catalytic reactions, future work could incorporate coverage-dependent activation energies derived from DFT calculations.

Despite these limitations, the combined DMC–RSM methodology provides a powerful, cost-effective approach to map intrinsic kinetics and identify promising operating windows before committing to resource-intensive high-pressure experiments. The framework is general and can be applied to other catalysts, feedstocks, and reaction conditions.

The intrinsic kinetic parameters extracted here, free from transport limitations and complex coverage effects present at elevated pressures, serve as an ideal foundation for building a hierarchical microkinetic model. Such a model, parametrized by the rate constants in Table 1 and augmented with pressure-dependent lateral interactions from DFT, would allow for the direct prediction of turnover frequencies at industrially relevant pressures—a task we are currently undertaking.

## 5 Conclusions

In this work, we resolved a long-standing mechanistic controversy in surface chemistry by demonstrating that methanol adsorbs on Ni(100) through both molecular and dissociative pathways—a finding that neither experimental nor computational approaches alone could previously establish. Using a DMC–RSM integrated framework rigorously validated against experimental TPD spectra (*R*^2^ = 0.9897) and independent isothermal data, we showed that only a dual-pathway model reproduces all experimental desorption profiles, providing the first conclusive evidence for the simultaneous operation of both channels. Systematic model comparison using AIC and BIC criteria confirmed that the improvement in fit is not due to overfitting but reflects the true requirement of the dual-pathway mechanism. Mechanistically, the dissociative pathway rapidly supplies surface D atoms responsible for early D_2_ and HD formation, while the molecular pathway provides a reservoir of CH_2_OD* that decomposes at higher temperatures, explaining the complex H_2_ desorption features. A quadratic RSM model identified temperature as the dominant factor, achieving an optimal hydrogen yield of 95.5% at 379.7 K, 3.50 µPa, and 107.8 s. Sensitivity analysis confirmed the kinetic robustness of our conclusions, with <1.5% yield variation under ±20% parameter perturbation. Beyond process optimization, the validated kinetic network establishes a quantitative framework for rational catalyst design—identifying the C–H scission of methoxy (DRC = 0.72) as the dominant rate-controlling step and suggesting strategies such as alloying or doping to selectively promote the dissociative pathway. Our findings advance fundamental understanding of alcohol reactivity on transition metal surfaces and provide a reliable intrinsic kinetic network for hierarchical microkinetic modeling, bridging the gap between surface science and catalytic engineering.

## Author contributions

Parveneh Kaboutari: conceptualization, methodology, software, validation, formal analysis, investigation, data curation, writing – original draft, visualization. Hadis Bashiri: conceptualization, methodology, resources, writing – review & editing, supervision, project administration, funding acquisition.

## Conflicts of interest

The authors declare no conflicts of interest.

## Supplementary Material

RA-016-D6RA06909F-s001

## Data Availability

All data generated or analyzed during this study are included in this published article and its supplementary information (SI) file. The supplementary information is available alongside the manuscript. Supplementary information: Fig. S1: lattice size convergence test. Fig. S2: cross-validation against independent isothermal experiments. Fig. S3: pareto analysis chart. Table S1: degree of rate control (DRC) analysis. Table S2: CCD design matrix with simulated yield data. Table S3: local sensitivity analysis results. See DOI: https://doi.org/10.1039/d6ra06909f.
