# Supplementary material for: Resolving the dual-pathway adsorption mechanism of methanol on Ni(100) via dynamic Monte Carlo simulations and optimizing hydrogen yield with response surface methodology
Source: RSC Adv. 2026 Sep 10;16(43):54000–8. doi: 10.1039/d6ra06909f (PMC13560877; doi:10.1039/d6ra06909f)
Supplement: Supplementary file 1 [file RA-016-D6RA06909F-s001.pdf]

Supplementary Information (SI) for RSC Advances.
This journal is © The Royal Society of Chemistry 2026

**Supplementary Information**

# Resolving the Dual-Pathway Adsorption Mechanism of Methanol on Ni(100) via Dynamic Monte Carlo Simulations and Optimizing Hydrogen Yield with Response Surface Methodology

Parveneh Kaboutari, Hadis Bashiri*

*Department of Physical Chemistry, Faculty of Chemistry, University of Kashan, Kashan, Iran*

Corresponding author. Fax: +98 31 55912397.

E-mail address: hbashiri@kashanu.ac.ir (H. Bashiri).

---

**Table S1.** Degree of rate control (DRC) for key elementary steps at the optimal conditions (T = 379.7 K, P = 3.50 μPa, t = 107.8 s).

DRC values were computed using the approach of Campbell [S1], where $\sum DRC_i = 1$. A value close to 1 indicates a dominant rate-controlling step.

| **Elementary step** | **DRC** |
|---|---|
| $CH_3O^* \rightarrow CH_2O^* + H^*$ (Step 3) | 0.72 |
| $CH_3OD(g) \rightarrow CH_3O^* + D^*$ (Step 2) | 0.11 |
| $CH_2O^* \rightarrow CHO^* + H^*$ (Step 5) | 0.06 |
| $H^* + H^* \rightarrow H_2(g)$ (Step 14) | 0.04 |

| Elementary step | DRC |
|---|---|
| Other steps (sum) | <0.07 |

**Table S2. Central composite design (CCD) matrix and corresponding simulated hydrogen yield from DMC simulations.**

| Run | Coded A (T) | Coded B (P) | Coded C (t) | T (K) | P (μPa) | t (s) | $H_2$ Yield (%) |
|---|---|---|---|---|---|---|---|
| 1 | -1 | -1 | -1 | 320.3 | 1.81 | 72.2 | 87.2 |
| 2 | +1 | -1 | -1 | 397.7 | 1.81 | 72.2 | 92.1 |
| 3 | -1 | +1 | -1 | 320.3 | 4.19 | 72.2 | 88.8 |
| 4 | +1 | +1 | -1 | 397.7 | 4.19 | 72.2 | 93.0 |
| 5 | -1 | -1 | +1 | 320.3 | 1.81 | 107.8 | 88.1 |
| 6 | +1 | -1 | +1 | 397.7 | 1.81 | 107.8 | 93.3 |
| 7 | -1 | +1 | +1 | 320.3 | 4.19 | 107.8 | 89.3 |
| 8 | +1 | +1 | +1 | 397.7 | 4.19 | 107.8 | 93.1 |
| 9 | -1.68 | 0 | 0 | 300.0 | 3.00 | 90.0 | 84.7 |
| 10 | +1.68 | 0 | 0 | 400.0 | 3.00 | 90.0 | 95.8 |
| 11 | 0 | -1.68 | 0 | 350.0 | 1.00 | 90.0 | 90.9 |

| Run | Coded A (T) | Coded B (P) | Coded C (t) | T (K) | P (µPa) | t (s) | $H_2$ Yield (%) |
|---|---|---|---|---|---|---|---|
| 12 | 0 | +1.68 | 0 | 350.0 | 5.00 | 90.0 | 91.7 |
| 13 | 0 | 0 | -1.68 | 350.0 | 3.00 | 60.0 | 90.2 |
| 14 | 0 | 0 | +1.68 | 350.0 | 3.00 | 120.0 | 91.9 |
| 15 | 0 | 0 | 0 | 350.0 | 3.00 | 90.0 | 92.6 |
| 16 | 0 | 0 | 0 | 350.0 | 3.00 | 90.0 | 92.4 |

**Table S3.** Local sensitivity analysis results showing the effect of ±20% variation of each kinetic parameter on hydrogen yield at the optimal conditions.

| Parameter | -20% change | +20% change | Max Δ Yield |
|---|---|---|---|
| $E_a$ (Step 3) – $CH_3O^* \rightarrow CH_2O^* + H^*$ | -1.2% | +1.4% | 1.4% |
| $E_a$ (Step 2) – $CH_3OD(g) \rightarrow CH_3O^* + D^*$ | -0.8% | +0.9% | 0.9% |
| $E_a$ (Step 14) – $H^* + H^* \rightarrow H_2(g)$ | -0.5% | +0.6% | 0.6% |
| $E_a$ (Step 5) – $CH_2O^* \rightarrow CHO^* + H^*$ | -0.3% | +0.4% | 0.4% |
| Other parameters | <0.3% | <0.3% | <0.3% |

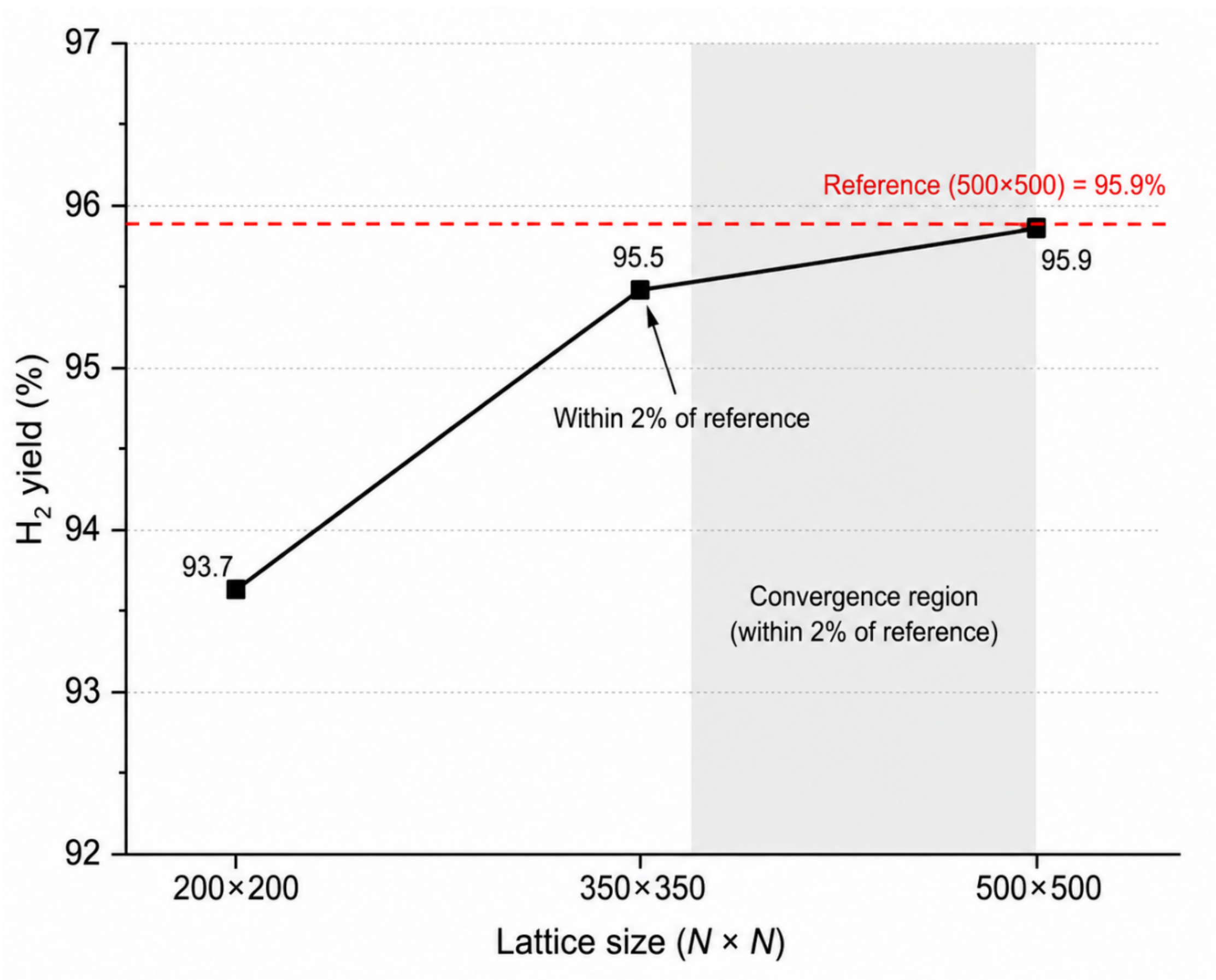


**Fig. S1. Lattice size convergence test.**

Hydrogen yield at the optimal conditions (379.7 K, 3.50 μPa, 107.8 s) simulated for square lattices of varying size. The 350×350 lattice yields results within 2% of the 500×500 reference, confirming convergence.

| Lattice size | $H_2$ Yield (%) |
|---|---|
| 200 × 200 | 93.7 |
| 350 × 350 | 95.5 |
| 500 × 500 | 95.9 |

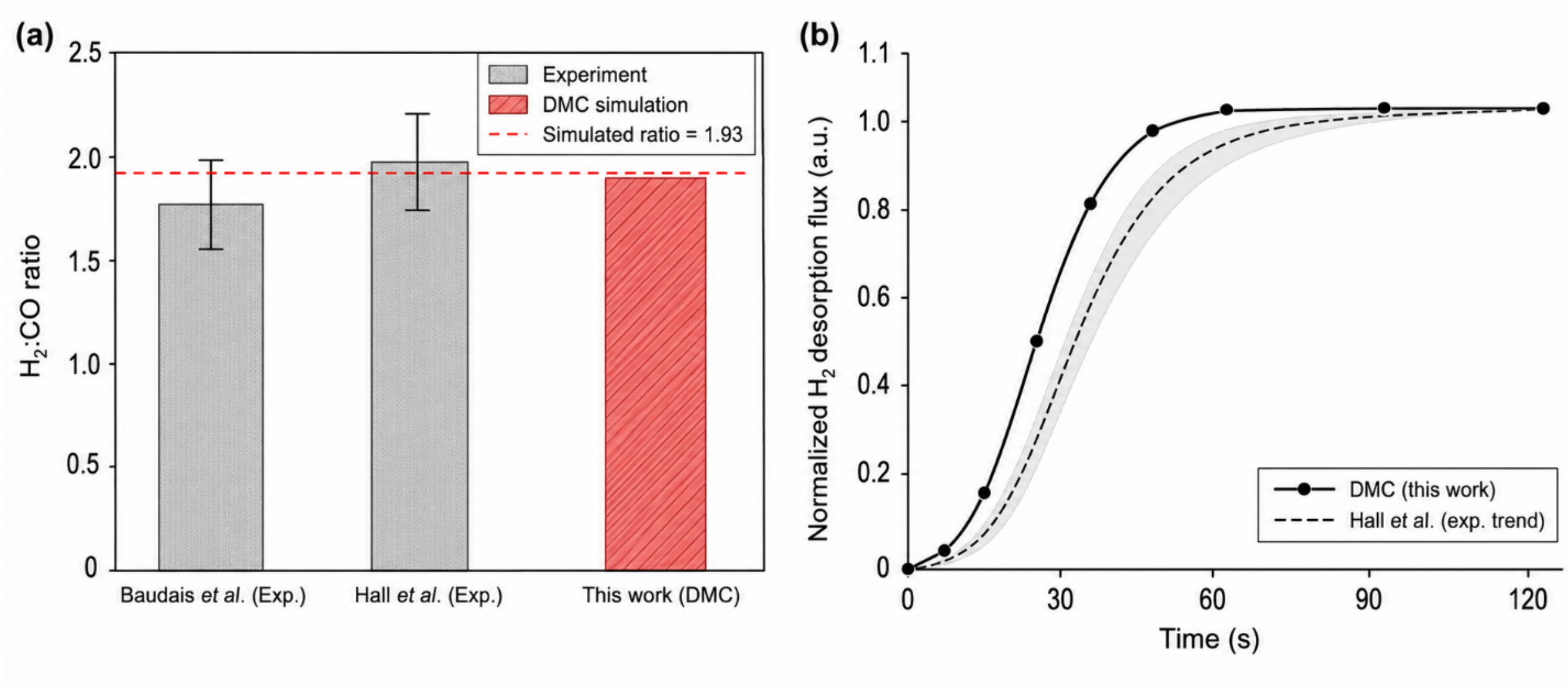


**Fig. S2. Cross-validation against independent isothermal experiments.**

**(a)** Comparison of simulated $H_2$:CO product ratio at 350 K and 3 μPa with data from Baudais et al. [27] and Hall et al. [32].

| Data source | $H_2$:CO ratio | This work (simulated) |
|---|---|---|
| Baudais et al. [27] | ~1.8 | 1.93 |
| Hall et al. [32] | ~2.0 | 1.93 |

**(b)** Time evolution of $H_2$ desorption flux at 350 K: simulation (solid line) versus laser-induced desorption trend from Hall et al. [32] (dashed line, normalized). The simulation reproduces the rapid initial rise and saturation behavior.

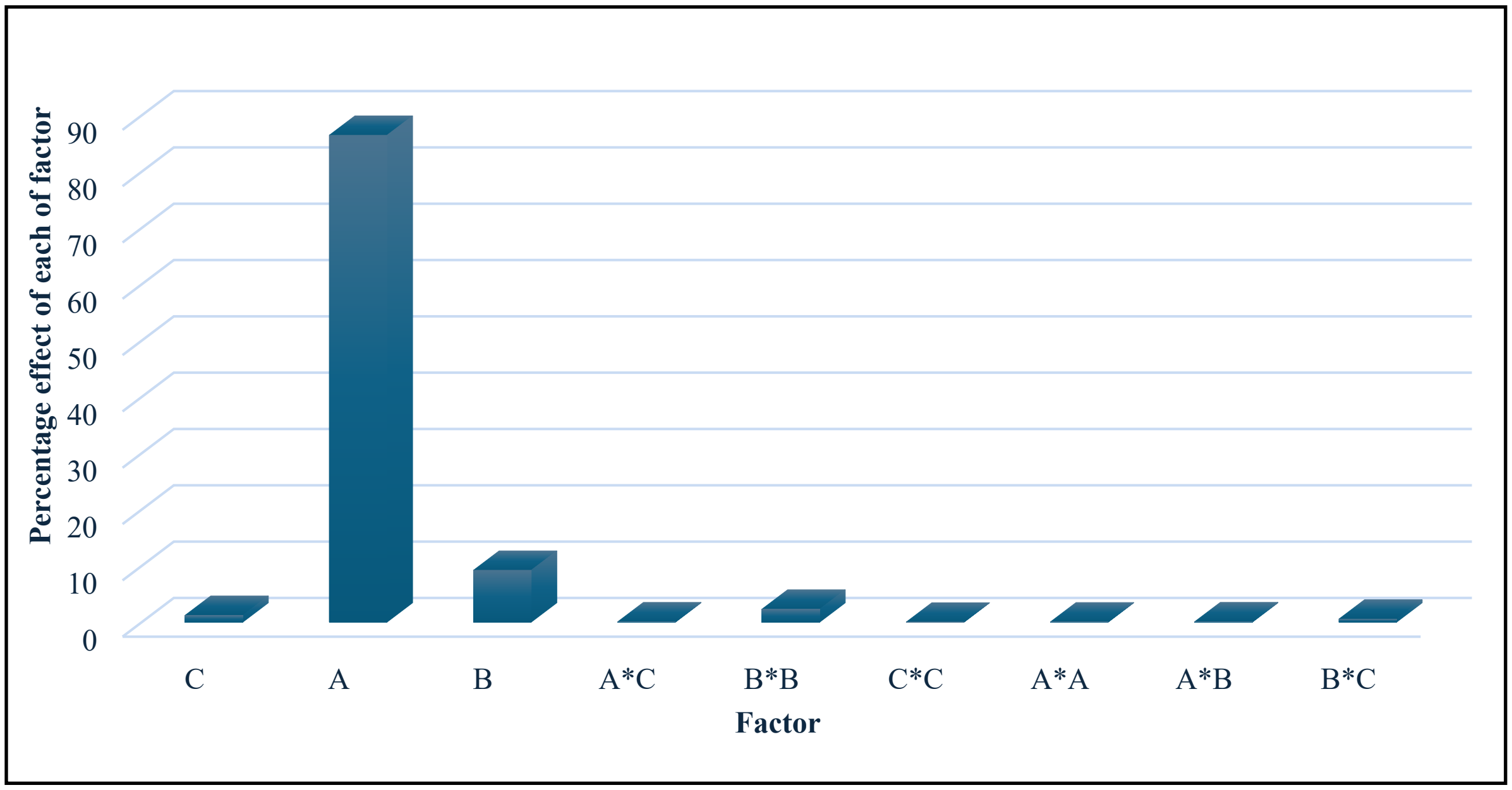


**Fig. S3. Pareto chart of standardized effects on hydrogen yield.**

Temperature (A) dominates with 86.5% of the total effect, followed by pressure (B, 9.2%), pressure quadratic effect ($B^2$, 3.8%), and time (C, 1.2%). Interaction terms (AB, BC) contribute less than 1% each.

---

**References for Supplementary Information**

[S1] C.T. Campbell, The degree of rate control: A powerful tool for catalysis research, J. Catal. 204 (2001) 520–524.

---

**END OF SUPPLEMENTARY INFORMATION**
